# Charcot Neuroarthropathy of the Foot and Ankle in the Acute Setting: An Illustrative Case Report and Targeted Review

**DOI:** 10.5811/westjem.59833

**Published:** 2023-08-30

**Authors:** Kian Bagheri, Albert T. Anastasio, Alexandra Krez, Lauren Siewny, Samuel B. Adams

**Affiliations:** * Campbell University, School of Osteopathic Medicine, Lillington, North Carolina; † Duke University Hospital, Department of Orthopedic Surgery, Durham, North Carolina; ‡ Duke University, School of Medicine, Durham, North Carolina; § Duke University Hospital, Department of Emergency Medicine, Durham, North Carolina

**Keywords:** Charcot neuroarthropathy, Charcot foot, diabetes mellitus, midfoot collapse, emergency

## Abstract

Charcot neuroarthropathy (CN) is a rare but serious sequela of diabetes and other diseases that cause peripheral neuropathy. It is most commonly characterized by degeneration of the foot and/or ankle joints leading to progressive deformity and altered weight-bearing. If left untreated, the deformities of CN lead to ulceration, infection, amputation, and even death. Because of the associated peripheral neuropathy and proprioception deficits that accompany CN, patients typically do not perceive the onset of joint destruction. Moreover, in the hands of the untrained clinician, the initial presentation of CN can easily be mistaken for infection, osteoarthritis, gout, or inflammatory arthropathy. Misdiagnosis can lead to the aforementioned serious sequelae of CN. Thus, an early accurate diagnosis and off-loading of the involved extremity, followed by prompt referral to a specialist trained in the care of CN are crucial to prevent the late-term sequelae of the disease. The purpose of this article was to create an opportunity for enhanced understanding between orthopedic surgeons and emergency physicians, to improve patient care through the optimization of diagnosis and early management of CN in the emergent setting.

## INTRODUCTION

Due to the progressive loss of protective sensation in advanced cases of diabetes mellitus (DM), identifying DM-related foot and ankle pathology in the early stages is a challenge for both patients and clinicians. One increasingly common complication of advanced DM is Charcot neuroarthropathy (CN), a progressive, destructive pathology of the bone and joints of the foot. Safavi et al estimated that CN has a prevalence of up to 13% in poorly controlled diabetic patients.^1^ Once thought to be a rare complication, this diagnosis has become increasingly common as the global incidence of DM continues to rise.

Diagnosing diabetic CN is challenging, and even the most experienced clinicians can struggle to differentiate the disease from pathologies that masquerade as CN. Limited specificity with regard to physical examination, imaging, and lab work findings in CN can cloud the diagnosis. Moreover, when CN presents as an acutely inflamed foot, it often presents similarly to much more common pathologies of the foot and ankle, such as cellulitis, osteomyelitis, or gout. Delayed treatment results in the development of foot and ankle deformities, which increases the risk of ulceration, infection, and amputation.^2^^–^^5^

This case illustration and review, authored by orthopedic surgeons and emergency physicians, focuses on the early clinical presentation, diagnosis, and management of CN, particularly in the emergent and outpatient setting. While many authors have provided review articles on CN,^2^^–^^5^ none to date have provided a manuscript specifically geared toward the facilitation of cross-talk between the involved specialties to aid in differentiating this challenging diagnosis from other similar pathologies. As the incidence of poorly controlled DM grows, the ability to distinguish the subtle presentation of CN from other more common foot pathologies takes on unprecedented importance. We advocate for enhanced collaboration between orthopedic surgeons and emergency physicians to better understand our respective roles in the early diagnosis and treatment of CN, and to ultimately improve the care of patients with this condition.

## ETIOLOGY AND PATHOPHYSIOLOGY OF CHARCOT NEUROARTHROPATHY

Early recognition of the complications of DM such as CN is critical in preventing damaging, long-term outcomes. In patients with poorly controlled DM, lack of protective sensation facilitates repetitive microtrauma, perpetuating a damaging feedback loop that is thought to contribute to the CN pathologic process. Some degree of CN exists in greater than 80% of patients who have poorly controlled DM for more than 10 years.^2^ However, it is essential to emphasize that diabetes alone is not the sole predisposing factor for the development of CN. Any condition that causes sensory or autonomic neuropathy can lead to CN. Examples include syphilis, in which degeneration and demyelination of the dorsal columns and roots can lead to progressive sensory ataxia, chronic alcoholism, in which nutritional deficiency (especially thiamine) and oxidative stress can lead to free radical damage to nerves,^6^ and spinal cord injury in which the somatosensory system is damaged.^7^

The exact pathogenesis of CN is not entirely understood, but there are two theories of what is believed to contribute to the pathogenesis of the disease.^2^ The neurovascular theory postulates that increased blood flow to the bones, which is a result of damage to the “trophic nerves,” leads to bone resorption and weakening. These pathophysiologic changes predispose patients to increased susceptibility to fractures and deformities. Alternatively, the neuro-traumatic theory states that repetitive trauma can cause fractures and deformation during healing.

## PATIENT CASE REPORT

A 75-year-old woman with longstanding, poorly controlled type 2 DM with peripheral neuropathy to the mid-calf region presented to the emergency department (ED) with complaints of right foot deformity and pain for nine days. While the patient did not recall any specific injury, she attributed the appearance and symptoms to an ankle sprain. She was initially evaluated by her primary care physician, where the diagnosis of cellulitis was made and she was told to go to the ED for further evaluation. At the time of presentation, her erythrocyte sedimentation rate (ESR) was 103, her white blood cell count (WBC) was within normal limits, and her vital signs were within normal limits. Physical exam revealed a swollen, diffusely erythematous foot with diminished sensation to fine touch. No standard foot and ankle radiographs were obtained, but she underwent magnetic resonance imaging (MRI) that showed a fluid collection posterior to the ankle joint, which was drained and yielded a clear, serous fluid that did not grow any organisms of culture. A diagnosis of cellulitis was made, and the patient was placed on intravenous clindamycin and admitted to the hospital. She was discharged on oral antibiotics five days later. There were no limitations placed on her weight-bearing status.

Shortly after discharge, the patient traveled across the country, bearing weight on the involved foot. After a week, her foot swelling and redness progressed in severity. She re-presented to a different ED. A repeat MRI at that time was concerning for osteomyelitis, and she was started on cefepime and vancomycin. During this presentation, her lab values were within normal limits and she was again afebrile. She was admitted to the hospital and later discharged on vancomycin via a peripherally inserted central catheter. No standard foot and ankle radiographs were obtained during this admission. Three weeks after that admission, her creatinine increased to 4.41 and her vancomycin trough was found to be 16.8 micrograms per microliter (mcg/mL) (reference range: 5–15 mcg/mL). Subsequently, the patient was admitted to the hospital, this time for acute renal failure secondary to vancomycin therapy.

**Figure 1.**
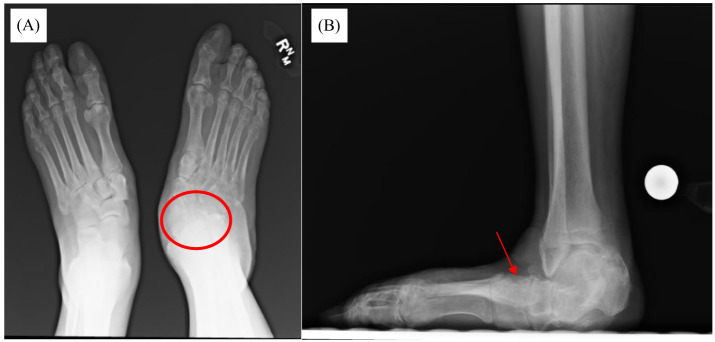
Weight-bearing radiographs: (A) Bilateral anterior-posterior. (B) Lateral radiographs 1.5 years after the patient’s initial presentation to the emergency department. There is evidence of degenerative changes at the talonavicular joint (A – red circle) in addition to midfoot collapse (B – red arrow).

**Figure 2.**
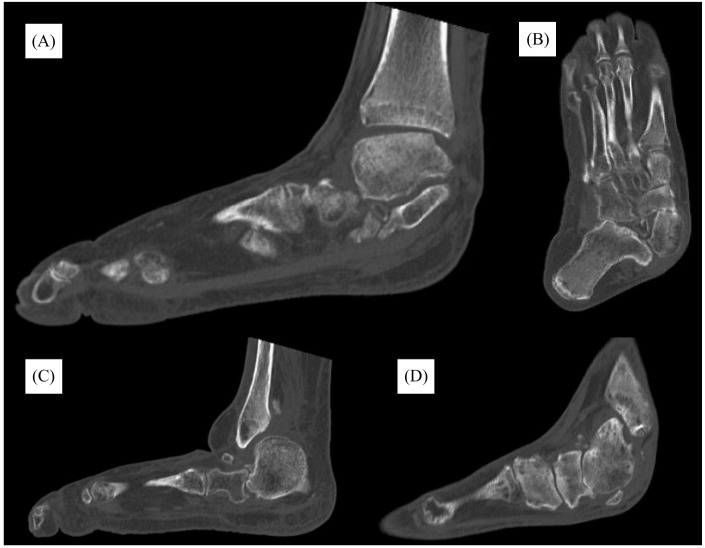
Computed tomography with (A,C,D) multiple sagittal cuts, and (B) a coronal cut after follow-up in orthopedic clinic demonstrating findings consistent with advanced stage Charcot joint with marked pes planus, mid-foot collapse, and hindfoot valgus.

Physical exam at the time revealed a fixed abduction deformity of the foot with a severe equinus contracture of the Achilles tendon. She did not have protective sensation to Semmes-Weinstein monofilament testing with 5.07. Finally, radiographic imaging was obtained, which demonstrated severe midfoot and hindfoot collapse with hindfoot valgus alignment and forefoot abduction (Figure 1). Her talus was nearly in a vertical plantar flexed position. Advanced imaging in the form of computed tomography (CT) further demonstrated these findings, with advanced stage CN, with degenerative change at the talonavicular joint and midfoot (Figure 2).

**Figure 3.**
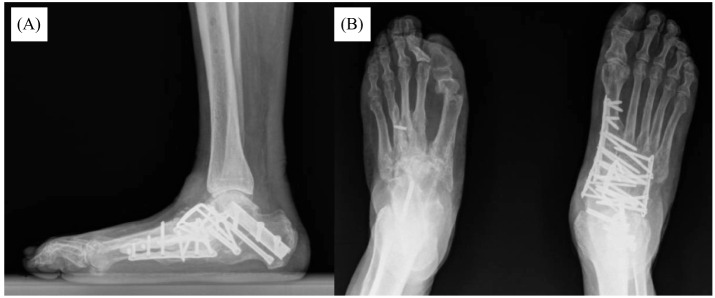
Most recent radiographs at final follow-up. (A) Lateral radiograph. (B) Bilateral anterior-posterior radiograph, revealing restoration of the arch of the foot, through the use of multiple osteotomy sites and internal fixation with a variety of plate, screw, and beam constructs.

She was eventually diagnosed with CN as well as end-stage renal disease, requiring chronic hemodialysis. After the correct diagnosis of CN had been made, the patient followed up in the orthopedic surgery clinic at our institution, where she received further care, including osteotomy with medial and lateral column fixation (Figure 3).

### Case Summation

This case highlights the importance of early recognition of CN in an acutely inflamed foot in patients with DM, especially in the presence of advanced neuropathy. Despite careful attention from various clinicians, no standard radiographs were obtained and the diagnosis of CN was missed, contributing to kidney injury in addition to increased destructive changes of the foot from CN that may have been avoided with prompt diagnosis. In the case of our patient, no individual clinician on the care team was at fault; CN can be a challenging diagnosis to make due to the number of pathologies with which it can be easily confused. Orthopedic surgeons receive specific training on differentiating CN from other conditions during foot and ankle subspecialty training, but the breadth of knowledge required for other specialties may preclude extensive awareness of CN. Thus, our goal with this review was to discuss the differential diagnosis of a patient with peripheral neuropathy and swelling to the foot and ankle and to expand upon the initial diagnosis and early treatment options of CN. Our ultimate aim is to facilitate understanding between orthopedic surgeons, emergency physicians, and primary care physicians to optimize the care of the diabetic patient with CN.

### Clinical Presentation in the Emergent Setting

The typical patient presenting to the ED with CN is an individual with poorly controlled diabetes, usually 50 years of age or older, with signs of advanced disease such as peripheral neuropathy, retinopathy, or chronic kidney disease. A clinician may encounter the frequent scenario of a patient with previous trauma to the foot, such as a fall, that re-presents weeks or months later with persistent swelling and pain. However, patients often do not recall any specific trauma, which makes prompt diagnosis challenging.

In the early phase of CN, pain is an uncommon finding. However, if present, pain is usually insidious in onset, progressing over days or weeks. Between 25–50% of patients recall no trauma or inciting event.^3^ A clinical exam will reveal unilateral warmth and an erythematous foot with soft tissue swelling. It is often difficult for the patient to fit the affected foot into normal shoes. A low threshold should be maintained for referral to orthopedic surgery.

Without treatment, early CN can progress to a chronic stage, where unfortunately most cases of CN are diagnosed. Late diagnosis increases the likelihood of bony destruction and resorption, which eventually leads to foot deformities, ulceration, and subsequent infection, often warranting amputation.^5^^,^^8^ In a systematic review by Safavi et al in 2021, the average time from symptom onset to a final diagnosis of CN was 84.8 days, and 48% of patients experienced a misdiagnosis.^1^ A systematic review by Korst et al in 2022 found similar results; the duration from symptom onset to correct diagnosis was 86.9 days, and 53.2% of cases of CN were initially misdiagnosed.^9^ These alarming statistics highlight the challenge in making the diagnosis of CN, which calls attention to the importance of accurate diagnosis and prompt treatment.

### Differential Diagnosis

Charcot neuroarthropathy shares many overlapping features with other common pathologies of the foot and ankle, which can complicate prompt and accurate diagnosis. Thus, we discuss common pathologies of the foot and ankle that can mislead clinicians to overlook the diagnosis of CN and offer clinical “pearls” that can help differentiate one from the other (Table 1).

**Table. 1:** A summary of risk factors, physical exam, laboratory, and imaging findings, and treatment for Charcot neuroarthropathy (CN) and diseases that present similarly to CN.

Differential	Risk factors	Physical exam findings	Abnormal lab findings from initial evaluation	Imaging findings	Treatment
Charcot neuropathy	• Chronic alcohol use • Diabetic neuropathy • Heavy metal poisoning • Myelomeningocele • Leprosy • Syphilis • Syringomyelia • Vitamin B12 deficiency	• Commonly painless • Decreased range of motion • Edema • Erythema that may decrease with elevation • Peripheral neuropathy • Structural deformity • Warmth	• Elevated HbA1c or random serum glucose	• Plain radiograph: Delayed imaging with evidence of joint space narrowing, fragmentation of articular surfaces, coalescence of fragments, bone resorption, soft tissue calcification, deformity and remodeling of the fracture fragments • MRI: May detect subtle changes above in the earlier stages as well as periarticular edema	• Contact casting • Orthotics • Optimize underlying risk factors • Surgical management
Gout	• Chemotherapy • Chronic kidney disease • Excessive intake of ethanol, dietary purine or fructose • Hemolytic disorders • Inherited enzyme defects leading to purine overproduction • Lymphoproliferative disorders • Male sex • Myeloproliferative disorders • Obesity • Older age • Psoriasis • Use of medications associated with hyperuricemia	• Decreased range of motion • Edema • Erythema • Fever • Joint pain • Joint tenderness • Subcutaneous nodules • Sudden onset of symptoms • Warmth	• Elevated serum uric acid • Joint aspiration with negative birefringent monosodium urate crystals	• Plain radiograph: Periarticular erosion with sclerotic borders, joint space narrowing, soft tissue crystal deposition	• Nonsteroidal anti-inflammatory drugs • Colchicine • Corticosteroids • Allopurinol • Optimize underlying risk factors
Deep vein thrombosis	• Chronic inflammatory states • Heart failure • Obesity • Older age • Male gender • Malignancy • May-Thurner syndrome • Myeloproliferative disorders • Pregnancy or postpartum status • Previous deep vein thrombosis • Prolonged immobilization • Recent trauma or surgery • Tobacco use • Use of oral contraceptives or hormone replacement therapy	• Edema • Erythema • Increased visible skin veins • Pratt’s sign • Sigg’s sign • Unilateral pain and edema most commonly in calf • Warmth	• Elevated D-dimer	• Ultrasound: Noncompressible vein positive for thrombus	• Anticoagulants • Optimize underlying risk factors
Osteoarthritis	• Female gender • History of joint trauma or overuse • Obesity • Older age • Muscle weakness	• Bone overgrowth or deformity • Decreased range of motion • Gradual onset of symptoms • Joint instability • Joint tenderness • May be polyarticular • Morning stiffness ≤30 minutes • Pain with movement and relieved with rest		• Plain radiographs: Joint space narrowing, presence of osteophytes, and subchondral sclerosis	• Nonsteroidal anti-inflammatory drugs • Physical therapy • Optimize underlying risk factors • Surgical management
Osteomyelitis	• Diabetes • Injection drug use • Immunocompromised • Indwelling catheter • Older age • Orthopedic hardware • Peripheral neuropathy • Poor vascular supply • Recent trauma • Sickle cell	• Decreased range of motion • Draining sinus tract • Gradual onset of symptoms • Fever • Erythema • Edema • Tenderness • Pain • Presence of ulcers • Warmth	• Elevated erythrocyte sedimentation rate and C-reactive protein Positive bone culture • Leukocytosis	• Plain radiograph: After 2 weeks may observe bone loss and periosteal reaction • MRI: In addition to finding on plain radiograph,T2 bone and soft tissue edema and a penumbra sign may be present	• Antibiotics • Irrigation and debridement • Optimize underlying risk factors • Amputation
Cellulitis	• Diabetes • Injection drug use • Lymphatic drainage disruption • Obesity • Peripheral artery disease • Recent surgery • Saphenous vein harvest • Skin barrier disruption • Skin inflammation • Venous insufficiency	• Breach in skin barrier • Edema • Erythema without dissipation upon elevation of affected extremity • Fever • Tenderness • Warmth	• Elevated erythrocyte sedimentation rate and C-reactive protein • Leukocytosis	-	• Antibiotics
Septic arthritis	• Diabetes • End-stage renal disease • Immunocompromised • Indwelling catheters • Injection drug use • Older age • Recent surgery • Recent trauma • Skin or soft tissue infection	• Acute onset of symptoms • Decreased range of motion • Edema • Erythema without dissipation upon elevation of affected extremity • Fever • Joint pain • Tenderness • Warmth	• Elevated erythrocyte sedimentation rate and C-reactive protein • Leukocytosis • Positive synovial fluid gram stain and culture	• Plain radiographs: Joint space narrowing, joint effusion, periarticular osteopenia, destruction of subchondral bone	• Antibiotics • Surgical drainage

*MRI*, magnetic resonance imaging.

### Infection: Cellulitis, Septic Arthritis, and Osteomyelitis

The diagnosis that is the most commonly made over CN is foot and ankle infection, including cellulitis, septic arthritis, and osteomyelitis. This is because acute CN causes foot swelling and erythema. In general, an acutely infected foot is often painful and accompanied by a fever, whereas CN may present with minimal pain and an afebrile status. However, varying presentations of more indolent infectious etiologies in afebrile patients who may lack peripheral sensation and pain symptoms can mislead the diagnosis. These patients also tend to have poorly controlled blood glucose levels due to elevated cortisol, which antagonizes the action of insulin,^10^ further causing the potential for misdiagnosis.

Charcot neuroarthropathy should always be considered in patients with recurrent cellulitis without systemic signs, symptoms, or findings that suggest infection on laboratory analysis.^3^ Specifically, cellulitis can be accompanied by signs of systemic toxicity such as leukocytosis and elevated inflammatory markers. The lack of these findings in the presence of erythema may indicate an early stage of CN. A common orthopaedic teaching point regarding the differentiation of CN from infectious etiologies involves assessing the limb for improvement in erythema with elevation. After the involved foot has been elevated for 30 minutes, erythema should decrease in CN but will remain in the setting of infection.^3^ Another distinguishing factor is laboratory markers. In CN, WBC count, C-reactive protein (CRP), and ESR may be normal, but these markers are almost always elevated to some extent in the setting of infection.^2^^,^^11^

Regarding specific infectious etiologies, osteomyelitis shares many overlapping features and can be concomitantly present with CN. Osteomyelitis should be suspected in the presence of ulcers and should be assumed in the setting of an ulcer that probes to bone.^12^ The ESR may be elevated in both osteomyelitis and CN, but a downward trend with appropriate antibiotic therapy favors the diagnosis of osteomyelitis.^13^ As a further differentiating factor, osteomyelitis often involves a single bone region, whereas CN involves multiple bones and joints.^14^

Imaging can further help delineate between CN and infectious etiologies of the foot and ankle. Plain radiography can be used to diagnose CN, but findings may not appear until two to three weeks after symptom onset. Moreover, it has been demonstrated that a 40–50% loss in bone mass in a patient with CN is required to note a difference from patients without CN.^12^ Thus, MRI is a reasonable alternative to plain radiography. Unfortunately, several limitations to MRI for the diagnosis of CN exist, such as in the setting of recent surgery, retained hardware, or renal insufficiency that may preclude patients from receiving contrast. A bone biopsy, which has long been considered the gold standard for diagnosing osteomyelitis, can be done after a 14-day antibiotic-free period if the diagnosis of osteomyelitis is more heavily favored than CN.^15^ In summation, differentiating CN from infectious etiologies of the foot and ankle is a clinical challenge that involves careful consideration of a variety of clinical, laboratory, and imaging-based findings.

### Gout

Gout most commonly manifests symptomatically in the foot and ankle, making this diagnosis easy to confuse with CN. Risk factors for gout and CN overlap, such as DM and obesity, but a few special considerations merit further discussion. For example, certain medications can precipitate gout, the most common of which are thiazide diuretics. Thus, we recommend a thorough medication reconciliation upon presentation in cases of suspected gout vs CN. Heavy alcohol use is another risk factor often associated with gout. However, chronic alcoholism has been shown to be a risk factor for the progression of CN,^16^^,^^17^ and so the presence of heavy alcohol use does not necessarily favor one diagnosis over the other. An additional diagnostic clue is the presence or absence of peripheral neuropathy. The presence of peripheral neuropathy coupled with extensive alcohol consumption points more toward CN over gout, especially if the patient does not have a previous history of gouty flares.^18^

To further differentiate a gout flare from CN, pain and tenderness that dissipate over a short duration favor the diagnosis of gout over the more long-term, subacute pain seen with CN. Laboratory analysis may reveal nonspecific changes consistent with inflammation during a gout flare, and these elevations may also be present in CN. Uric acid levels are not consistently elevated in gout^19^ and should not be used as a marker to distinguish gout from CN. If gout is suspected, synovial fluid testing with cell counts and differential white count, Gram stain and culture, and crystal examination under polarizing light microscopy is recommended.^20^^–^^22^ The presence of monosodium urate crystals confirms the diagnosis of gout.

### Deep Vein Thrombosis

Deep vein thrombosis (DVT), while more commonly relegated to the calf, may also present similarly to CN. One of the most reliable clinical signs of DVT is unilateral leg swelling that involves the calf.^23^ Risk factors for DVT include recent surgery, pregnancy, smoking, cancer, and prolonged immobility, which are distinct from CN.^24^ Furthermore, a D-dimer level has a high sensitivity for ruling out DVT. If the D-dimer is elevated, venous duplex ultrasonography becomes the imaging study of choice in patients with suspected DVT.^25^^,^^26^ The result of this study will be normal in patients with CN.

### Osteoarthritis

Osteoarthritis is a chronic condition that progressively worsens over the course of a patient’s life.^27^ Pain is the cardinal symptom of osteoarthritis, which may or may not always be present in CN, given loss of protective sensation. However, the pain in osteoarthritis is classically exacerbated by activity and improved by rest.^28^ Osteoarthritis also has characteristic findings on radiographs, such as joint space narrowing, the presence of osteophytes, and subchondral sclerosis.^29^^,^^30^ In addition, bony fragmentation and callus formation are not seen in osteoarthritis but can be seen in CN.

### Diagnosis

Diagnostic delays in diabetic CN are common but avoidable if clinicians maintain a high index of suspicion in patients with risk factors for CN. We encourage clinicians to always consider CN in their differential diagnosis when a diabetic patient with peripheral neuropathy presents with foot edema, redness, and warmth, especially in the absence of pain. Diagnosis of CN includes a thorough history and physical exam, imaging, and laboratory evaluation.

#### Physical Exam

Diagnosis of CN begins with a complete physical exam. Vital signs should be assessed for evidence of systemic illness, such as the presence of fever, tachycardia, or hypotension. In addition, the patient’s feet should be inspected for the presence of ulcers or breaks in the skin. Although gross sensation testing in patients at risk of neuropathy is simple and rapid in the emergent setting, it is unreliable for assessing diabetic patients at risk of CN.^31^ Semmes-Weinstein monofilament testing has long been considered the gold standard for detecting clinically relevant neuropathy in diabetic patients.^31^ This non-invasive, low-cost, and rapid test is the most sensitive indicator of CN.^32^ In addition to sensation testing, skin texture, bony deformities, signs of vascular or neurologic compromise, and details of footwear should be recorded.^2^ Finally, elevating the affected foot for 30 minutes is a simple yet effective way of narrowing the differential diagnosis in the emergent setting. Redness will decrease in CN but not in the setting of infection.^3^

#### Laboratory Testing

Laboratory testing in patients suspected of CN should include a complete blood count (CBC), WBC with differential, and testing of renal function (via blood urea nitrogen and creatinine). Patients should also be screened for diabetes, even if they do not have a history of the disease or its presenting risk factors. Depending on the clinical setting, this can be achieved by assessing fasting glucose, hemoglobin A1c (HbA1c), or random blood glucose levels. The ESR and CRP can also be helpful, as elevations in these values are seen more commonly in other conditions, such as osteomyelitis or infection.

#### Imaging

Imaging is also helpful to help establish the diagnosis of CN; however, it is imperative to emphasize that normal radiographs do not rule out CN. Radiographs are often unremarkable in the early stages of the disease and are not a reliable indicator of disease progression. Nonetheless, plain film radiographs must be obtained because they serve as a baseline and allow for the detection of subtle changes that may portend instability.^3^

In instances where the radiograph is negative, but there remains a high index of suspicion for CN, MRI can be used for further evaluation. Magnetic resonance imaging is more sensitive in detecting subtle changes in the early phase of the disease and can help rule out other overlapping pathologies such as osteomyelitis.^32^ In instances when MRI is contraindicated or cannot effectively distinguish CN from other pathologies, nuclear medicine studies such as leukocyte-labeled bone scans are highly specific for distinguishing between CN and infection.^33^ In this process, WBCs are removed from the patient, tagged with a radioisotope such as indium-111, and injected intravenously back into the patient. The tagged leukocytes then localize to areas of relatively new infection.

### Treatment

#### Immobilization

Charcot neuroarthropathy is a medical emergency that can lead to irreversible skeletal destruction and permanent deformities if not promptly addressed.^33^ In patients with concomitant risk factors for CN, immobilization and non-weight bearing of the involved foot is recommended until the correct diagnosis is confirmed. Once the diagnosis of CN is established, it is vital to continue immobilization and non-weight bearing of the affected extremity.^32^ At a minimum, the patient should be placed in a controlled ankle motion (CAM) boot. We caution against splinting or casting in the ED in case the patient gets lost to follow-up. A long-term splint or cast could lead to ulceration in the setting of decreased sensation. If an ulcer is present, the preferred method of immobilization is a total contact cast.^3^^,^^33^ Full methodology for the application of total contact casting is outside the scope of this manuscript but generally includes a thin layer of cast padding to allow for weight distribution to offload points of ulceration and bony change. Total contact casting should continue until lower extremity edema and warmth resolve and serial radiography shows evidence of osseous consolidation.^3^ This process typically occurs after three to four months but can take as long as 12 months. A CT or MRI can also be used to monitor the resolution of CN.

## DISCUSSION

Charcot neuroarthropathy continues to emerge as a significant contributor to patient morbidity worldwide. In recognition of the importance of early diagnosis of this condition, a number of authors have reported on various interventions aimed to improve the detection of CN. A study by Foltz et al looked at the vascular and neurologic findings that were the most helpful in differentiating factors between patients with and without CN.^34^ They found that simple neurologic testing combined with a comprehensive patient history were the most beneficial tools to determine patients at high risk of developing CN. They found statistically significant associations for history of retinopathy, nephropathy, and previous foot ulcer in addition to the neurologic physical exam findings of vibratory sensation, deep tendon reflexes, and Semmes-Weinstein monofilament testing with CN. Notably, vascular examinations were not predictive of CN. These findings have important implications for emergency physicians who are considering a diagnosis of CN.

Another study by Chantelau et al assessed the clinical course of acute CN in 24 patients without evidence of definite fractures on the initial radiograph after the onset of symptoms.^35^ Alarmingly, 19 of the 24 patients in this study had been misdiagnosed prior to referral, highlighting the difficulty in achieving an accurate diagnosis of CN. The most common misdiagnosis among these patients was an ankle sprain, followed by cellulitis. Fourteen of the patients progressed to definite fractures of either all tarsometatarsal joints or of the talonavicular joint. This study emphasizes the importance of early diagnosis, treatment, and referral of CN to prevent the progression of the disease.

If CN is promptly diagnosed, early treatment has shown excellent success at mitigating its possible long-term ramifications. A study by Parisi et al in 2013 looked at the radiographic and functional results in the treatment of the early stages of CN with a CAM boot and immediate weight-bearing.^36^ The study included 22 patients with type 2 diabetes. American Orthopedic Foot and Ankle Society scores showed statistically significant improvement at the termination of the study. These authors conclude that the utilization of a CAM boot in conjunction with immediate weight-bearing for CN showed good functional outcomes and halted the progression of deformity on radiographic assessment. In contrast, when the diagnosis of CN is delayed and patients present to the ED with the presence of a Charcot-related foot wound, outcomes can be much worse. A study by Wukich et al found that the presence of a Charcot-related foot wound at presentation increased the likelihood of a major lower extremity amputation by a factor of six.^37^

## CONCLUSION

Charcot neuroarthropathy is a highly complex disorder, and its pathophysiology is not fully understood. While once a rare diagnosis, as obesity and diabetes rates rise, CN is becoming more common. Even experienced clinicians may struggle to diagnose CN, given the large number of other conditions that can present similarly. It should always be on the differential in a diabetic patient with peripheral neuropathy and signs of foot edema, redness, and warmth, especially in the absence of pain. If CN is suspected, immediate immobilization and non-weight bearing are recommended until orthopedic follow-up. A multidisciplinary approach should be emphasized in the care of patients with CN, with enhanced collaboration between emergency physicians and orthopedic surgeons to ensure the best outcome for patients with this complex condition.
